# Impact of oxygen concentration on time to resolution of spontaneous pneumothorax in term infants: a population based cohort study

**DOI:** 10.1186/1471-2431-14-208

**Published:** 2014-08-23

**Authors:** Huma Shaireen, Yacov Rabi, Amy Metcalfe, Majeeda Kamaluddeen, Harish Amin, Albert Akierman, Abhay Lodha

**Affiliations:** 1 Department of Pediatrics, University of Calgary, Foothills Medical Centre, Rm C211, 1403-29TH Street, T2N 2 T9 Calgary, Alberta, Canada; 2 Alberta Health Services, Calgary, Alberta, Canada; 3 Alberta Children’s Hospital Research Institute, Calgary, Alberta, Canada; 4 Department of Obstetrics & Gynecology, University of Calgary, Calgary, Canada; 5 Community Health Sciences, University of Calgary, Calgary, Canada

**Keywords:** Oxygen, Pneumothorax, Newborn and nitrogen wash out

## Abstract

**Background:**

Little evidence exists regarding the optimal concentration of oxygen to use in the treatment of term neonates with spontaneous pneumothorax (SP). The practice of using high oxygen concentrations to promote “nitrogen washout” still exists at many centers. The aim of this study was to identify the time to clinical resolution of SP in term neonates treated with high oxygen concentrations (HO: FiO_2_ ≥ 60%), moderate oxygen concentrations (MO: FiO_2_ < 60%) or room air (RA: FiO_2_ = 21%).

**Methods:**

A population based cohort study that included all term neonates with radiologically confirmed spontaneous pneumothorax admitted to all neonatal intensive care units in Calgary, Alberta, Canada, within 72 hours of birth between 2006 and 2010. Newborns with congenital and chromosomal anomalies, meconium aspiration, respiratory distress syndrome, and transient tachypnea of newborn, pneumonia, tension pneumothorax requiring thoracocentesis or chest tube drainage or mechanical ventilation before the diagnosis of pneumothorax were excluded. The primary outcome was time to clinical resolution (hours) of SP. A Cox proportional hazards model was developed to assess differences in time to resolution of SP between treatment groups.

**Results:**

Neonates were classified into three groups based on the treatment received: HO (n = 27), MO (n = 35) and RA (n = 30). There was no significant difference in time to resolution of SP between the three groups, median (range 25th-75th percentile) for HO = 12 hr (8–27), MO = 12 hr (5–24) and RA = 11 hr (4–24) (p = 0.50). A significant difference in time to resolution of SP was also not observed after adjusting for inhaled oxygen concentration [MO (a HR = 1.13, 95% CI 0.54-2.37); RA (a HR = 1.19, 95% CI 0.69-2.05)], gender (a HR = 0.87, 95% CI 0.53-1.43) and ACoRN respiratory score (a HR = 0.7, 95% CI 0.41-1.34).

**Conclusions:**

Supplemental oxygen use or nitrogen washout was not associated with faster resolution of SP. Infants treated with room air remained stable and did not require supplemental oxygen at any point of their admission.

## Background

Pneumothorax is one of the most common air leak syndromes that occurs in the newborn period [1]. It is classified into primary pneumothorax (without any obvious lung diseases) and secondary pneumothorax (due to underlying lung pathology, or associated with precipitating factors such as transient tachypnea of newborn, meconium aspiration, continuous positive pressure ventilation (CPAP), mechanical ventilation, pneumonia, respiratory distress syndrome or post surfactant treatment) [1-5]. Spontaneous pneumothorax (SP) is a form of primary pneumothorax in neonates. It usually occurs in the absence of inciting risk factors at birth [1]. The mechanism is related to maladaptive transition after birth. The presence of persistently high or unequal transpulmonary inflating pressure in the alveoli during the transition period results in rupture of alveoli into the pleural space and produces a spontaneous pneumothorax [6,7]. The incidence of radiologic SP is 1% to 2% and symptomatic SP is 0.05% to 1% in all live births [1,4,8]. Pneumothorax increases morbidity, prolongs hospital stay, causes parental anxiety and, in some cases, can also result in death [1,8,9].

The adult literature suggests that inhaling higher concentrations of oxygen between 60% to 100% (nitrogen washout) compared to room air improves the rate of resolution of symptomatic SP [10-12]. The theory of nitrogen washout proposes that the inhalation of 100% oxygen reduces the partial pressure of nitrogen in the alveolus compared to the pleural space. This gradient difference causes the nitrogen to diffuse from the pleural space into the alveoli, resulting in resorption of air from the pleural space into the alveoli and faster resolution of the pneumothorax [6,10]. The treatment of SP in term neonates is based on theoretical and historical hypotheses, which favor the use of higher oxygen concentrations/nitrogen washout for rapid resolution of SP [13,14]. However, there is minimal published evidence available for the optimal inspired oxygen concentration requirement to treat clinically significant SP in term neonates [15-17]. Empirically, SP is treated with variable concentrations of oxygen [17].

Treatment of SP with higher oxygen concentrations may lead to hyperoxic injury in neonates [18]. Hyperoxia produces free oxygen radicals and enhances cellular apoptosis. It can alter the genetic expression of the cell and may be a cause of potential childhood cancers [19]. Unrestricted oxygen use poses a financial burden to the health care system due to prolonged neonatal intensive care unit (NICU) admission [15]; this burden is more profound in resource poor countries [16].

To the best of our knowledge, in the term neonatal population, no standardized management guidelines or concrete evidence are available in the literature that show an advantage or disadvantage of using higher concentrations of oxygen for resolution of spontaneous pneumothorax. In our NICU there are three practice patterns. Some neonatologists treat SP with nitrogen washout (60 to 100% inspired O_2_ concentration); some titrate the O_2_ concentration targeting a pulse oximeter O_2_ saturation (SpO_2_) ≥ 95%; and some treat with room air alone. We hypothesized that term neonates who inhaled higher oxygen concentrations/nitrogen washout would have a quicker resolution of spontaneous pneumothorax as compared to infants treated with room air or lower oxygen concentrations. The aim of this study was to identify the time to clinical resolution of spontaneous pneumothorax in term neonates treated with high fraction of inspired oxygen (FiO_2_)/nitrogen washout (HO: FiO_2_ ≥ 60%), moderate oxygen (MO: FiO_2_ < 60%) or room air (RA: FiO_2_ = 21%).

## Methods

### Data source and settings

This was a population based cohort study of spontaneous pneumothorax in term neonates in Calgary, Alberta, Canada. We reviewed the medical records (both electronic data and patients’ charts) of all term infants admitted with a diagnosis of pneumothorax to all NICUs in Calgary between 1st January 2006 to 31st December 2010. The list of patients was retrieved from the NICU database and the medical records departments by identifying infants with an ICD-9-CM (512 and 512.81) and an ICD-10-CM (J93.1 and P25.1) code for pneumothorax. Ethics approval was obtained from the Conjoint Health Research Ethics Board at the University of Calgary.

All term newborns (gestational age ≥37 weeks) admitted to the NICU from birth to 72 hours of life with signs of respiratory distress and a diagnosis of spontaneous pneumothorax confirmed by chest X-ray (CXR), were included in the study. Newborns with congenital/chromosomal anomalies, history of meconium stained liquor/meconium aspiration, respiratory distress syndrome, transient tachypnea of newborn, pneumonia, tension pneumothorax requiring thoracocentesis or chest tube drainage, those who received positive pressure ventilation (PPV) or mechanical ventilation (MV) before the diagnosis of spontaneous pneumothorax were excluded.

Based on initial fraction of inspired oxygen concentration used at admission for the treatment of SP, neonates were divided into three groups: high fraction of inspired oxygen (FiO_2_)/nitrogen washout (HO: FiO_2_ ≥ 60%), moderate oxygen (MO: FiO_2_ < 60%) or room air (RA: FiO_2_ = 21%). The decision to treat a patient with room air, high or moderate oxygen concentration was based on individual attending neonatologist preference.

The initial concentration of oxygen used for treatment of spontaneous pneumothorax was the primary exposure variable. Duration of oxygen therapy was calculated from the initiation of oxygen therapy until clinical resolution of pneumothorax or till treatment failure (development of tension pneumothorax within 72 hours of diagnosis and treatment of SP). Data on maximum O_2_ concentration inspired during the treatment period was based on the highest recorded dose documented in the chart. The method of oxygen delivery at the time of admission was either via oxyhood, or nasal cannula. The concentration of inspired oxygen via oxyhood was analyzed through an oxygen analyzer. The concentration of inspired oxygen through nasal prongs was calculated according to the “STOP- ROP effective FiO_2_ conversion table” [20], a modified equation described by Benaron and Benitz [21]. The severity of respiratory distress was classified according to the validated “Acute care of at-risk newborn (ACoRN)” respiratory score [22-24]. The estimation of pneumothorax size was abstracted from the radiologist report; radiologists were blinded to the treatment group. There was no validated method available to measure the size of pneumothorax on CXR in neonates so an estimation was made according to adult guidelines as mentioned in the British Thoracic Society (BTS) guidelines [10], and from the neonatal literature [8,25-27].

Clinical resolution of pneumothorax was defined as cessation of respiratory distress and discontinuation of oxygen treatment with maintenance of SpO_2_ ≥ 95%. Nitrogen washout was defined in our NICU as the use of 60 to 100% of inspired O_2_ continuously for at least 6 hours.

Time to clinical resolution of spontaneous pneumothorax, measured in hours was the primary outcome variable. Neonates were followed from admission until clinical resolution of pneumothorax as documented in the patient chart. The secondary outcome variables were length of hospital stay for the treatment of pneumothorax and treatment failure (development of tension pneumothorax within 72 hours of diagnosis and treatment of SP). Tension pneumothorax was characterized by rapid instability of vital signs and shifting of the mediastinum on CXR, requiring thoracocentesis, chest tube insertion or mechanical ventilation [25].

### Statistical analysis

Descriptive statistics were used to describe the study population. Chi-square tests, analysis of variance (ANOVA) and Kruskal-Wallis tests were used to assess differences in categorical and continuous variables stratified by the concentration of oxygen received. A Cox proportional hazards model was used to assess differences in time to clinical resolution of SP. A crude model and a model adjusted for infant gender and ACoRN respiratory score were derived. An adjusted hazard ratio (aHR) >1 would indicate a benefit of oxygen treatment. P value < 0.05 was taken as significant. All analyses were conducted in Stata SE version 11.

## Results

**Figure 1:**
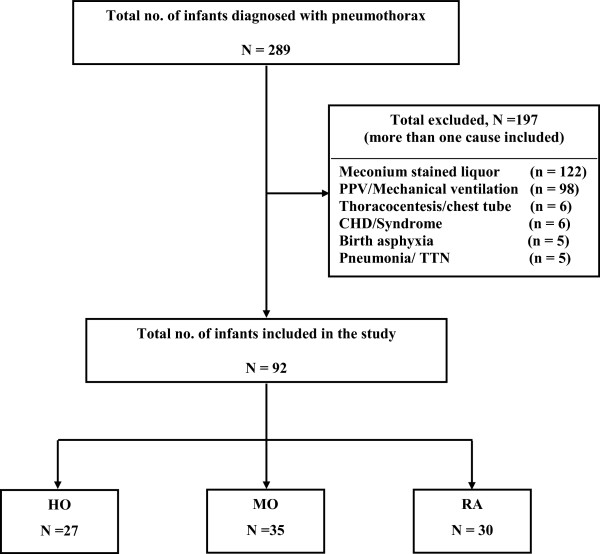
**Flow diagram showing population profile.** RA = room air, MO = moderate oxygen concentration, HO = high oxygen concentration, PPV = positive pressure ventilation, CHD = congenital heart disease, TTN = transient tachypnea of newborn.

Two hundred and eighty nine medical charts of neonates, admitted between 1st January, 2006 to 31st December, 2010 with a diagnosis of pneumothorax were reviewed (Figure 1). Ninety two neonates had spontaneous pneumothorax and were included in the study. Based on a total number of 80,819 births during the study period, the incidence of pneumothorax was 0.35% and that of SP was 0.11% at our centre.

Eligible neonates were further classified into 3 groups, according to the inhaled oxygen concentration at the initiation of treatment; 27 in the HO group (FiO_2_ ≥60%), 35 in the MO group (FiO_2_ < 60%) and 30 in the RA group (FiO_2_ 21%). All neonates received oxygen via an oxyhood in the HO group. In the MO group, 30 neonates received oxygen via an oxyhood and 5 neonates received blended oxygen through nasal prongs. There was no cross over in the treatment groups. All the patients in the HO, MO and RA groups remained in their designated groups. The maternal baseline characteristics were similar in all three groups (Table 1). Neonatal baseline characteristics in terms of gestational age, birth weight, resuscitation requirement at birth, Apgar scores and admission age were comparable. Although male infants were more likely to be treated with RA than MO or HO (p = 0.01), this observation could be due to chance and be a reflection of the small sample size (Table 1). This difference was adjusted for in the model (a HR = 0.87, 95% CI 0.53-1.43, p = 0.59).

**Table 1:** Baseline maternal and neonatal characteristics

**Characteristics**	**RA (n = 30)**	**MO (n = 35)**	**HO (n = 27)**	***P***
**Maternal**				
Maternal age (yrs), mean (SD)	29.4 (5.4)	28.6 (5.9)	30.0 (6.2)	0.64
Antenatal corticosteroids, n (%)	0 (0.0)	2 (5.7)	0 (0.0)	0.19
Chorioamnionitis, n (%)	3 (10.0)	2 (5.7)	2 (7.4)	0.81
Cesarean section, n (%)	8 (26.7)	16 (45.7)	13 (48.1)	0.18
**Neonatal**				
Male, n (%)	28 (93.3)	24 (68.6)	16 (59.3)	0.01
GA, wks, mean (SD)	39 (1.2)	39 (1.2)	39 (1.2)	0.90
BW, g, mean (SD)	3328.5 (471.3)	3480.9 (487.8)	3405.0 (435.7)	0.43
Resuscitation at birth, n (%) (free flow oxygen only)	4 (13.3)	8 (22.9)	5 (18.5)	0.62
Apgar score at 5 min, median (range 25th -75th percentile)	9 (8–9)	9 (9–9)	9 (8–9)	0.41
Age of admission, hours, median (range 25th -75th percentile)	0.3 (0.2-1.0)	1 (0.1-20.0)	0.5 (0.2-3.0)	0.19

RA = room air, MO = moderate oxygen concentration, HO = high oxygen concentration, SD= standard deviation, GA = gestational age, BW = birth weight.

**Figure 2:**
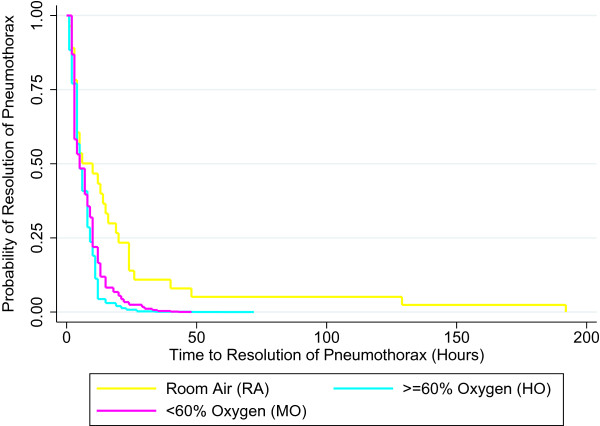
Survival curve of time to clinical resolution of pneumothorax by concentration of oxygen received (adjusted model).

The median (range 25%-75%) time to clinical resolution of SP was 11 hours (4–24) for infants treated with RA, 12 hours (5–24) for infants treated with MO and 12 hours (8–27) for infants treated with HO (p = 0.5). Both crude (MO HR = 0.84, 95% CI 0.50-1.43; RA HR = 1.06, 95% CI: 0.64-1.76) and adjusted (for infant sex and ACoRN respiratory score) [MO (a HR = 1.13, 95% CI 0.54-2.37, p = 0.75); RA (a HR = 1.19, 95% CI 0.69-2.05, p = 0.52)] models did not indicate a statistically significant difference in the time to resolution of spontaneous pneumothorax based on treatment group, indicating that treatment with different concentrations of inhaled oxygen do not significantly alter the hazard function and influence time to resolution of SP. This is supported by the overlapping survival curves between the 3 treatment groups found in Figure 2. All groups experienced a comparable resolution time of approximately 12 hours for the majority of infants, with occasional neonates requiring treatment for approximately 24 hours. Exceptional neonates requiring prolonged treatment due to underlying disease were observed in all treatment groups. Two neonates in the RA group had prolonged time to resolution of pneumothorax (130–160 hours), one had pneumothorax associated with sepsis and the other had pneumothorax and subcutaneous emphysema. In the MO (2 neonates) and HO (4 neonates) groups, prolonged time to resolution (48–60 hours) was associated possibly with delayed pulmonary adaptation to the extra uterine life. We speculate those neonates might have underlying labile pulmonary hypertension. Those neonates did not require mechanical ventilator support, inhaled nitric oxide and their blood gases were normal.

To maintain oxygen saturations more than 95%, the inspired oxygen concentration at initiation of treatment was also higher in the HO group. Neonates in the HO group were sicker, had higher ACoRN scores (Table 2) and their oxygen saturations were also low at the time of admission to the NICU (Table 1). However, the adjusted hazard ratio after adjustment with ACoRN score (a HR = 0.74, 95% CI 0.41-1.34, p = 0.32) did not show significant difference in the time to clinical resolution of SP (Figure 2). Six neonates in total (MO (n = 4/35) and HO (n = 2/27)) had treatment failures (tension pneumothorax, thoracocentesis, chest tube placement or mechanical ventilator support), and they were excluded from the analysis. They might have more serious underlying disease, though the p-value was not significant (Table 2). While in RA group, none of the neonate developed tension pneumothorax or treatment failure. Two neonates from MO and HO group had chest tube drains followed by thoracocentesis, while the other two neonates in MO group only required thoracocentesis. Ventilator support was required by one neonate in HO and 2 in MO group. An important observation was that none of the neonates in the RA group required supplemental oxygen treatment at any time of their hospital stay. The length of NICU stay was similar in all three groups (Table 2). There were no deaths observed in any group.

**Table 2:** Neonatal outcomes

**Characteristics**	**RA (n = 30)**	**MO (n = 35)**	**HO (n = 27)**	***P***
**Median (range 25th-75th percentile)**				
SpO_2_ (%) at admission	96.5 (94–99)	89 (81–94)	88 (83–92)	< 0.001
Concentration of oxygen (%) inspired at initiation of treatment	21 (21–21)	35 (29–40)	85 (65–100)	< 0.001^a^
Maximum oxygen concentration (%) inspired during the treatment period	21 (21–21)	40 (30–50)	85 (65–100)	< 0.001^a^
Total duration of supplemental oxygen therapy, hours	0 (0–0)	10 (3–23)	12 (8–27)	0.19^a^
Respiratory rate at admission	63 (52–75)	65 (54–74)	72 (64–80)	0.13
ACoRN score at admission	3 (2–4)	4 (3–6)	6 (5–7)	< 0.001
ACoRN score according to severity				< 0.001
<5	26 (86.7)	23 (65.7)	2 (7.4)	
≥5	4 (13.3)	12 (34.3)	25 (92.6)	
Length of hospital stay, hours	47.3 (23.5-64.0)	47.3 (24.0-95.3)	47.5 (27.3-71.9)	0.54
**Number (%)**				
Site of pneumothorax on chest x-ray				0.88
Right	11 (36.7)	17 (48.6)	13 (48.1)	
Left	10 (33.3)	10 (28.6)	7 (25.9)	
Bilateral	9 (30.0)	8 (22.9)	7 (25.9)	
Size of pneumothorax based on CXR				0.18
Small	21 (70.0)	25 (71.4)	15 (55.6)	
Moderate	9 (30.0)	6 (17.1)	10 (37.0)	
Large	0 (0.0)	4 (11.4)	2 (7.4)	
Failure of treatment	0 (0.0)	4 (11.4)	2 (7.4)	0.17^a^

SpO_2_ = saturation of peripheral oxygen. ^a^Wilcoxon Rank Sum Test was used to assess statistical differences. P-values only represent a comparison between the HO and MO groups.

## Discussion

Symptomatic spontaneous pneumothorax is one of the main reasons for admission of term newborn infants to the NICU. As most cases of SP present with mild respiratory distress, the management of SP with or without supplemental oxygen is invariably different between physicians [16,17]. No consensus exists regarding the treatment of symptomatic spontaneous pneumothorax in stable term neonates. Questions such as whether or not spontaneous pneumothorax in term neonates should be treated with supplemental oxygen and whether higher oxygen concentrations, based on the historical hypothesis of nitrogen washout is helpful in the speedy resolution of spontaneous pneumothorax are still unanswered. To the best of our knowledge, this is the first study which focuses on the time to clinical resolution of SP in neonates treated with room air (21% FiO_2_) and with different supplemental oxygen concentrations.

No difference was observed in the time to clinical resolution of SP between infants treated with room air or different concentrations of oxygen in our study. Even after adjustment for respiratory morbidity and gender, neonates in the HO group (≥60% FiO_2_) did not show a rapid resolution of the SP.

There are several studies on neonatal pneumothorax and its risk factors; however there is a scarcity of studies on the effect of oxygen dose dependant resolution of SP in the neonatal population. An older study by Yu et al. in 1975 noted speedy resolution of spontaneous pneumothorax (within 48 hours) in neonates treated with higher oxygen concentrations [14]. The drawback of this study is that it included both term and preterm neonates with all types of pneumothorax; and there was no comparison done with respect to different oxygen concentrations on the resolution rate of SP. A more recent study examined the time to resolution of spontaneous pneumothorax with 100% O_2_ inhalation in 45 term and near term neonates (>35 weeks gestation age) [17]. In this retrospective study, Clark et al. compared neonates receiving inhaled 100% O_2_ (nitrogen washout group) (n = 26) and neonates receiving different concentrations of O_2_ targeting the SpO_2_ between 92 to 95% (n = 19), conventional therapy group [17]. Their findings corroborate our results. They did not find a significant difference in the mean time to resolution of tachypnea (20 hours, standard deviation (SD) ± 26 vs. 37 hours, SD ± 27, p = 0.181), mean length of hospital stay (3.53 days, SD ± 1.68 vs. 4.35 days, SD ±1.96, p = 0.168) and mean time to first oral feed between the conventional therapy and nitrogen washout groups [17]. Our study differs in that we studied the impact of different O_2_ concentrations as well as the effect of room air (21% FiO_2_) on the resolution of SP. We observed that the time to resolution of SP was not significantly longer in neonates who were just in room air (21% FiO_2_) vs. neonates receiving different O_2_ concentrations higher than the room air. An interesting finding was that neonates in room air never required supplemental oxygen, or experienced treatment failure (pneumothorax, need for thoracocentesis, chest tube insertion or mechanical ventilation) at any time during their hospitalization. This could mean that room air (21% FiO_2_) may be as effective as any higher inhaled O_2_ concentrations for resolution of SP. Future trials will possibly find the room air as the optimal O_2_ concentration requirement for resolution of symptomatic pneumothorax and prevention of O_2_ toxicity.

All other studies which focused on the resolution rate of spontaneous pneumothorax in association with inhaled O_2_ concentration were conducted on animal subjects. A randomized clinical trial on 23 rabbits, in whom unilateral pneumothorax was induced, Ronald et al. observed [28] that the resolution rate of pneumothorax was shorter (36 hours) in the group that received a higher concentration of oxygen (FiO_2_ ≥ 60%), compared to the group that was treated with room air (48 hours) [28]. In two other studies on rabbits by England [29] and Zierold [30], the rate of resolution was dependent on the concentration of oxygen. The higher the concentration of inspired oxygen, the faster the resolution of pneumothorax. Two studies were identified in adults for the treatment of pneumothorax with oxygen inhalation [11,31]. A prospective study in adults by Chadha [31] observed an increased rate of resolution of pneumothorax when treated with higher concentrations of inspired oxygen. The other study in 22 adult patients, divided into two groups (1-treated with room air and 2- intermittently treated with room air and oxygen from 9–36 hours, at 16 L/min flow) also showed a positive correlation on the rate of absorption of pneumothorax with oxygen treatment [11]. The resolution rate of pneumothorax was 4.8 cm^2^/day with room air in both groups and increased to 17.9 cm^2^/day with oxygen treatment in the second group [11]. No side effects of oxygen therapy were observed in this small study.

This retrospective study has few limitations due to the selection and information bias (detection bias). It was difficult to control bias and confounders due to the absence of randomization and blinding in this study. We had no control over some variables, therefore for homogeneity and as a reference we recorded these variables when these were observed at the time of admission. Although the use of higher oxygen concentration in sick neonates in HO and MO groups seemed justifiable at the admission, there was no targeted SpO_2_ goal observed to wean the inspired O_2_ concentrations despite of the decrement in respiratory severity scores. The oxygen concentration used at admission and the duration of oxygen treatment was widely variable and depended on the discretion of the admitting physician. The prescription for continuous “nitrogen washout” for minimum of 6 hours was clearly documented in the nursing notes, however there was no indication mentioned for titration of O_2_ concentration on achievement of SpO_2_ > 95%. This reflects the cultural and historical beliefs of physicians and nurses with regard to nitrogen washout treatment. We observed a decreasing trend in the use of oxygen treatment over time; while this did not achieve statistical significance (p = 0.822), this may be related to the small sample size when stratified by oxygen concentration and year. This decreasing trend may be the influence of emerging new literature on oxygen treatment as a drug and its pros and cons, especially toxicity of oxygen free radicals.

We did not have a CXR on every patient at the time of discharge from the NICU to determine the radiologic resolution of pneumothorax. Therefore we decided to record the time to clinical resolution of SP from clinical notes in the patient charts. According to the BTS guidelines [10], chest computed tomography (CT) scan is the best modality for accurate estimation of the size of pneumothorax. However, the size of pneumothorax does not always correlate well with the severity and resolution of symptoms associated with the pneumothorax [10]. Therefore, the management should be tailored according to the clinical severity [10]. The same clinical judgment for pneumothorax treatment is also applied to neonatal management. In newborns, there is no validated method available for correct estimation of size of pneumothorax on CXR. Portable CT scans are very costly, not available for sick babies and carry the risk of radiation. Due to the above mentioned reasons and inconsistency of the radiological reporting for the size of pneumothorax in neonates, the significance of size with the time to resolution of pneumothorax is not very reliable. Therefore, for the purpose of our study, we determined that the best option for calculating the time to resolution of pneumothorax was the clinical resolution of signs. The results of our study are applicable in neonates at other centres.

## Conclusions

This study has been the first to determine the time to clinical resolution of spontaneous pneumothorax in term neonates treated with room air and different concentrations of inhaled oxygen. We found that supplemental oxygen use was not associated with faster resolution of SP in HO and MO groups. Neonates who were treated with room air rather than supplemental oxygen did not have longer recovery times. Oxygen treatment for pneumothorax should be viewed as a prescribed drug, with documentation for its indication, target saturation and titration goals. Future prospective trials for the treatment of symptomatic spontaneous pneumothorax using 100% inspired oxygen concentration vs. room air in sick neonates with targeted SpO_2_ goal, will be beneficial in developing an acceptable treatment guideline for term neonates.

## Consent

This was a retrospective study based on the chart review. Personal information of patients was not disclosed. A waiver of the consent was obtained from the Conjoint Health Research Ethics Board at the University of Calgary.

## Abbreviations

BTS: British Thoracic Society; CXR: Chest X-ray; CT: Computed tomography; CPAP: Continuous positive pressure ventilation; SP: Spontaneous pneumothorax; SpO2: Saturation of peripheral oxygen; FiO2: Fractional inspired oxygen; SD: Standard deviation.

## Competing interests

The authors declare that they have no competing interests.

## Authors’ contributions

HS. Has conceptualized and designed the study, drafted the initial manuscript, and approved the final revised manuscript as submitted. YR. Analyzed the results, reviewed and revised the manuscript, and approved the final revised manuscript as submitted. AM. Designed the statistical model, carried out the data analyses, reviewed and revised the manuscript, and approved the final revised manuscript as submitted. MK. Critically reviewed and revised the manuscript, and approved the final revised manuscript as submitted. HA. Critically reviewed and revised the manuscript, and approved the final revised manuscript as submitted. AA. Critically reviewed and revised the manuscript, and approved the final revised manuscript as submitted. AL. Conceptualized, helped in the designed study, critically reviewed and revised the manuscript from inception to the final manuscript. All authors read and approved the final manuscript.

## Pre-publication history

The pre-publication history for this paper can be accessed here:

http://www.biomedcentral.com/1471-2431/14/208/prepub
